# Is Age-targeted full-field digital mammography screening cost-effective in emerging countries? A micro simulation model

**DOI:** 10.1186/2193-1801-2-366

**Published:** 2013-07-31

**Authors:** Fabiano Hahn Souza, Carísi Anne Polanczyk

**Affiliations:** Institute for Health Technology Assessment (IATS), Porto Alegre, RS Brazil; Graduate Studies Program in Epidemiology, School of Medicine, Federal University of Rio Grande do Sul, Porto Alegre, RS Brazil; Radiology and Oncology Department of the State of São Paulo Cancer Institute, University of São Paulo, Medical School, São Paulo, SP Brazil

## Abstract

**Objective:**

The present paper estimates the cost-effectiveness of population-based breast cancer (BC) screening strategies in Brazil for women under 50 years from the perspective of the Brazilian public health system.

**Methods:**

A Markov model, simulating the natural history of female BC sufferers in Brazil, was developed. This model compares the lifetime effects, costs, and cost-effectiveness of seven BC screening strategies in women between 40 to 49 years: (A) usual care; (B) annual screen-film mammography (SFM); (C) SFM every 2 years; (D) annual full-field digital mammography (FFDM); (E) FFDM every 2 years; and (F and G) age-targeted options, with FFDM annually until 49 years and SFM annually (or biannually) from 50 to 69 years.

**Results:**

Adopting SFM every 2 years (Strategy C) was found to be slightly more costly but also more effective in terms of quality-adjusted life years (QALYs), yielding an incremental cost-effectiveness ratio (ICER) of R$ 1,509 per QALY gained. Annual SFM (Strategy B) was the next best option at an additional R$ 13,131 per QALY gained. FFDM annual screening (Strategy E) was dominated by Strategy F, the age-targeted option. For younger women, the age-based strategy had an ICER of R$ 30,520 per QALY gained. In the sensitivity analysis, the ICERs ranged from R$ 15,300 to R$ 257,899 in different regions of the country, depending on BC incidence, population age distribution, and mammography coverage.

**Conclusions:**

SFM every 2 years for all women starting between the ages of 40 and 49 would be a cost-effective strategy. Taking into account regional specificities, age-targeted FFDM is one option to improve the outcomes of BC patients in an emerging country.

## Introduction

Breast cancer (BC) is the most frequently diagnosed cancer and the leading cause of cancer among females, accounting for 23% of total cancer diagnoses and 14% of overall cancer deaths (Jemal A et al. 2011). Moreover, BC is now the leading cause of cancer-related death among females in developing countries, a shift from the previous decade when cervical cancer was the most common cause of cancer-related death. Although cancer incidences and patterns differ according to level of human development, female BC is the only type of cancer that is common in all regions of the world. Thus, the global control of BC through both early detection and primary prevention is a high priority (Bray et al. 2012). Specifically in the context of this study, there is a high incidence of BC in the female population in Brazil with more than 50 new cases diagnosed per 100,000 women every year (INCA 2008).

Major advances in the early diagnosis of some cancers and a better understanding of the pathogenesis of the disease have led to risk reduction and prevention strategies. These advances as well as improvements in therapy have all contributed to declines in cancer-related death rates (Jemal et al. 2008). However, these successes have come with substantial increases in cost, causing a serious financial burden on patients, families, and society at large (Meropol et al. 2009). Currently, the most effective method for preventing premature mortality and morbidity due to BC is the increased use of screening programs and adjuvant therapies (Berry et al. 2005). In particular, effective early detection strategies are preferred to adjuvant therapies because they result in less morbidity.

For the past 30 years, conventional screen-film mammography (SFM) has been the method of choice for the radiological evaluation of the breast (Tabar & Dean 2008). The demonstration of the efficacy of mammography in reducing BC mortality by approximately 15% in younger women (<50 years) (Nelson et al. 2009) led to recommendations in some countries to introduce routine screening programs for this subgroup (Schopper & de Wolf 2009). However, considerable controversy over whether screening is effective for women aged 40–49 years has halted the adoption of a broad screening approach. Further, because SFM has lower sensitivity mainly due to the greater breast density and higher rates of tumor growth in younger women (Buist et al. 2004), full-field digital mammography (FFDM) has been shown to be superior to SFM in this subgroup (Souza et al. 2013; Pisano et al. 2008; Skaane et al. 2007).

FFDM is based on a different technology, in which each exposure produces a digital image (Tice & Feldman 2008). Although BC age-targeted screening (digital for women <50 years) is reasonably cost-effective in the US (Tosteson et al. 2008), no studies of the cost-effectiveness of FFDM screening in younger women have yet been carried out in middle-income countries. The objective of this study is thus to explore the cost-effectiveness of population-based BC screening using different strategies for women aged 40–49 years in the Brazilian public health system.

## Material and methods

### Mathematical model

**Figure 1:**
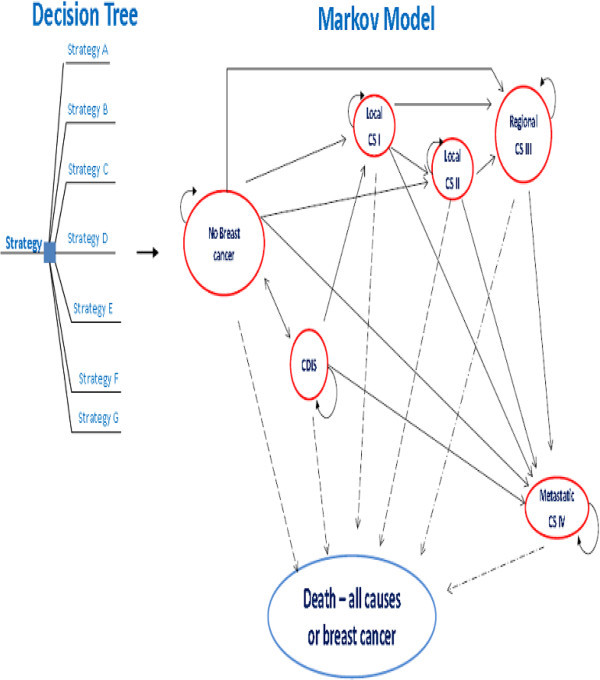
**The disease process model for breast cancer.**

The developed mathematical model was constructed using decision analysis software (TreeAgePro2009 Suite, release 1.0.2, Tree Age Software, Inc., Williamstown, MA). Specifically, a Markov model was used to compare populations of young women in Brazil. The structure of the model (Figure 1) is similar to other models used for BC screening programs and characterizes the complexity of the natural history of the disease (e.g., invasive stages are defined following the tumor–node–metastasis classification (Edge et al. 2010; van Oortmarssen et al. 1990; Szeto & Devlin 1996; Rojnik et al. 2008).

In the real world, women diagnosed with BC have a relatively high risk of developing a new cancer or a recurrent disease. Women who develop a recurrent disease will create extra cost and utilities for the state in line with the basal risk of recurrence of the higher Markov state.

The micro simulation approach with a cycle length of 1 year with half-cycle correction was chosen for this study. BC incidence, mammography sensitivity, mortality, and relative survival rate were modeled as time-dependent transition probabilities.

We considered the following seven BC screening strategies for women aged 40–49 years: (A) usual care; (B) annual SFM; (C) SFM every 2 years; (D) annual FFDM; (E) FFDM every 2 years; (F) “age-targeted digital” (i.e., annual FFDM for the 40–49 age group and annual SFM for the 50–69 age group); and (G) “age-targeted digital” (annual FFDM for the 40–49 age group and SFM every 2 years for the 50–69 age group). These strategies were based on the findings of previous studies (Schopper & de Wolf 2009; US Preventive Services Task Force 2009) and they included the current status of the Brazilian public health system (Strategy A “no formal BC screening” as the base case). According to DATASUS, the Brazilian public health system database, the annual utilization of SFM is approximately 17.5% in women above 50 years (Ministério_Saúde_Brasil, DATASUS 2011).

To determine whether the increased costs of screening strategies are warranted by health gains compared with usual care, we assessed the cost-effectiveness of these seven screening strategies from a public healthcare perspective. The time horizon covered the full lifetimes of the sample population from age 40 onward. Mammography screening stops after 69 years as recommended by the Brazilian National Cancer Institute (INCA) (INCA 2007). The starting age for the micro simulation ranged from 40 to 49 years based on the Brazilian population census (IBGE 2010).

### Model calibration

Data on age-dependent cancer incidence were obtained from the Population-based Cancer Registry in Brazil (INCA 2010). The clinical-stage distributions for usual care and screening strategies were used from INCA (INCA 2011) and from the literature, respectively. Table 1 presents the main parameters used in the model.

**Table 1:** **Main parameters used in the base case and sensitivity analyses**

Variables	Screening test performance			Distribution/comments	Reference
	Mean	Minimum	Maximum		
Mammography coverage	18%	10%	30%	Uniform	(Ministério_Saúde_Brasil, DATASUS 2011)
Mammography coverage ^ǁ^	70%	55%	85%	Uniform	(Lilliu et al. 2002)
Sensitivity of SFM (40–49 years)	76%	60%	85%	Effectiveness data from large population	(Kerlikowske et al. 2011)
Sensitivity of FFDM (40–49 years)	82%	65%	90%	Effectiveness data from large population	(Kerlikowske et al. 2011)
Sensitivity of SFM (50–59 years)	85%	65%	90%	Effectiveness data from large population	(Kerlikowske et al. 2011)
Sensitivity of FFDM (50–59 years)	80%	65%	90%	Effectiveness data from large population	(Kerlikowske et al. 2011)
Sensitivity of SFM (60–69 years)	83%	65%	90%	Effectiveness data from large population	(Kerlikowske et al. 2011)
Sensitivity of FFDM (60–69 years)	90%	65%	95%	Effectiveness data from large population	(Kerlikowske et al. 2011)
Treatment complication (yearly) - Chemotherapy	16%	10%	20%	Resource utilization database	(Hassett et al. 2006)
Treatment complication (yearly) – Endocrine therapy	5%	1%	10%	Resource utilization database	(Hassett et al. 2006)
Overdiagnosis	5%	0	30%	Systematic review estimate	(Smith & Duffy 2011)
	**Cancer stage distribution**				
	**Mean**	CI^*^95%			
DCIS (clinical diagnostic)	6.1%	4.9–7.3%		Beta (α = 97; β = 1494)	(INCA 2009b; Martins et al. 2009)
State 1 (clinical diagnostic)	14%	13.1–16.6%		Beta (α = 232; β = 1329)	(INCA 2009b; Martins et al. 2009)
State 2 (clinical diagnostic)	38.6%	36.5–40.5%		Beta (α = 915; β = 1455)	(INCA 2009b; Martins et al. 2009)
State 3 (clinical diagnostic)	34.7%	32.4–37.1%		Beta (α = 546; β = 1028)	(INCA 2009b; Martins et al. 2009)
State 4 (clinical diagnostic)	10.8%	NA		Complementary	(INCA 2009b; Martins et al. 2009)
CDIS (screening diagnostic)	6.1%	NA		Dynamic range ^∫^	(Kerlikowske et al. 2011)
State 1 (screening diagnostic)	58%^Ξ^	NA		Effectiveness data from large population	(Kerlikowske et al. 2011)
State 2 (screening diagnostic)	32.4%^Ξ^	NA		Effectiveness data from large population	(Kerlikowske et al. 2011)
State 3 (screening diagnostic)	8.3%^Ξ^	NA		Effectiveness data from large population	(Kerlikowske et al. 2011)
State 4 (screening diagnostic)	1.3%^Ξ^	NA		Effectiveness data from large population	(Kerlikowske et al. 2011)
	**Transition probabilities**				
**BC Recurrence**	**Mean**	**Range**	**Local**	**Regional/systemic**	
CDIS	0.008/y	0.002–0.014/y	50–98%	2–50%	(Baxter et al. 2004; Meijnen et al. 2008)
Stage 1	0.030/y	NA	16–47%	53–84%	(Hirsch et al. 2011a; Hirsch et al. 2011b)
Stage 2	0.087/y	NA	19–56%	44–81%	(Wapnir et al. 2006)
Stage 3	0.283/y	0,11–0,28/y	19–56%	19–56%	(Wapnir et al. 2006)
**BC Death**	**Mean**	**Range**			
CDIS	0.002/y	0.002–0.003/y			(Ernster et al. 2000)
Stage 1	0.009/y	NA			(de Oliveira et al. 2009)
Stage 2	0.031/y	NA			(de Oliveira et al. 2009)
Stage 3	0.090/y	NA			(de Oliveira et al. 2009)
Stage 4	0.270/y	0.20–0.34			(de Oliveira et al. 2009)
	**Relative risk**		**Distribution/comments**		
	**Mean**				
Adjuvant Taxane chemotherapy^§^	0.86		Log-Normal (μ = −0.15;σ=0.07)		(Peto et al. 2012)
Adjuvant Aromatase inhibitor^§¶^	0.82		Log-Normal (μ = −0.20;σ=0.12)		(Dowsett et al. 2010)
Adjuvant Trastuzumab therapy ^§‡^	0.61		Log-Normal (μ = −0.49;σ=0.06)		(Perez et al. 2011)
Screening vs. non-screening cancer cases^Φ^	0,62		Log-Normal (μ= − 0.48;σ=0.12)		(Mook et al. 2011)
Advanced disease - Luminal A vs. Luminal B^¥^	1.42		Log-Normal (μ = 0.34;σ=0.12)		(Kennecke et al. 2010)
Advanced disease - Luminal A vs. HER2 + ^¥^	1.90		Log-Normal (μ=0.64;σ=0.11)		(Kennecke et al. 2010)
Advanced disease - Luminal A vs. Triple negative^¥^	1.62		Log-Normal (μ = 0.48;σ=0.11)		(Kennecke et al. 2010)
	**Relative odds ratio**		**Distribution/comments**		
	**Mean**				
Diagnostic cancer downstage (FFDM under 50 years)	0.54		Log-Normal (μ= − 0.654;σ=0.307)		(Souza et al. 2013)
	**Mean**	**Minimum**	**Maximum**		
Discount rate	5%	0%	10%	Brazilian Health Economic Guidelines	(Ministério_Saúde_Brasil 2009)
**Costs (Brazilian Real)**					
	**Mean**	**Minimum**	**Maximum**		
Medical visit	10	5	25	DATASUS	(Ministério_Saúde_Brasil, DATASUS 2011)
FFDM	68	45	90	Estimated^∀^	(Souza 2012)
SFM	45	30	60	DATASUS	(Ministério_Saúde_Brasil, DATASUS 2011)
Biopsy	429	150	700	Gamma (α = 14.93; λ = 0.03)	(Souza 2012)
Recall SFM	152	50	250	Aggregate costs	(Souza 2012)
Recall FFDM	197	100	300	Aggregate costs	(Souza 2012)
Staging early BC^Ψ^	509	250	750	Gamma (α = 3.09 λ=0.01)	(Souza 2012)
Staging locally and advanced cancer^Δ^	592	200	800	Gamma (α = 2.52 λ=0.04)	(Souza 2012)
Invasive cancer stage 1 (first year)	6,502	2,500	11,500	Aggregate costs	(Souza 2012)
Invasive cancer stage 2 (first year)	15,610	6,500	24,500	Aggregate costs	(Souza 2012)
Invasive cancer stage 3 (first year)	18,638	9,500	27,500	Aggregate costs	(Souza 2012)
Invasive cancer stage 4 (first year)	12,452	6,500	20,500	Aggregate costs	(Souza 2012)
Invasive cancer stage 1 (≥ 2 year)	602	200	1,000	Aggregate costs	(Souza 2012)
Invasive cancer stage 2 (≥ 2 year)	677	200	1,200	Aggregate costs	(Souza 2012)
Invasive cancer stage 3 (≥ 2 year)	742	200	1,600	Aggregate costs	(Souza 2012)
Invasive cancer stage 4 (≥ 2 year)	12,439	4000	20,000	Aggregate costs	(Souza 2012)
	**Utilities**				
	**Mean**	CI^*^95%			
Healthy woman	0.800	NA		South of Brazil population^⊥^	(Cruz 2010)
Healthy woman – false positive mammography	0.795	NA		Estimated^∴^	(Cruz 2010)
Non metastatic BC^χ^ – follow-up	0.772	0.63–0.90		Normal distribution	(Souza 2012; Cruz 2010)
Early BC^χ^ – Adjuvant Endocrine Therapy	0.762	0.62–0.91		Normal distribution	(Souza 2012; Cruz 2010)
Early BC^χ^ – Adjuvant Chemotherapy	0.739	0.61–0.87		Normal distribution	(Cruz 2012; Cruz 2010)
Clinical Stage 3 – Adjuvant Endocrine Therapy	0.760	0.59–0.95		Normal distribution	(Souza 2012; Cruz 2010)
Clinical Stage 3 – Adjuvant Chemotherapy	0.700	0.63–0.78		Normal distribution	(Souza 2012; Cruz 2010)
Clinical Stage 4 – Advanced disease	0.680	0.57–0.80		Normal distribution	(Souza 2012; Cruz 2010)

^ǁ^ Screening strategies; NA: not applicable; ^∫^ time and screening coverage-dependent (increase in the DCIS rate with the introduction of the screening program); ^Ξ^ relative to invasive cancer (excluding DCIS); ^§^ Relative risk of BC death in clinical stage 2 and 3 patients; ^¶^ hormone-positive patients; ^‡^ HER2-positive patients; ^Φ^ Relative risk of BC death; ^¥^ Relative risk of BC death in advanced disease (stage 4) according to prognostic subtype; ^∀^ Plausible estimate 50% above SFM reimbursement value; ^Ψ^ clinical stages 1 and 2; ^Δ^ clinical stages 3 and 4; ^*^ confidence interval; ^⊥^ Porto Alegre city; ^∴^Considering the mean of non-metastatic BC utility (0.77) and a false positive as a 2-month period of disutility (0.80–0.77= [(0.03)*(0.16 year)=0.005] →0.80–0.005=0.795; ^χ^*in situ*, stage 1, stage 2, and stage 3 patients.

Transitions to Markov states are governed by the rate of incidence, clinical-stage distribution data, and sojourn time. We modeled for an increase in the incidences of in situ carcinomas through the introduction of screening mammography (Kerlikowske 2010). Ten years after the introduction of these screening programs, ductal carcinoma in situ (DCIS) incidence rates were assumed to have stabilized.

Moreover, evidence of better prognostic screening compared with the pre-screening era for in situ cancer cases was incorporated into our model (Ernster et al. 2000), while over diagnosis was adjusted for confounding and lead time bias according to the findings of Smith and Duffy (Smith & Duffy 2011). The risks and benefits of screening schedules were also adapted from Mandelblatt et al. (Mandelblatt et al. 2009). BC prognosis subgroups were then determined for advanced disease status: Luminal A, Luminal B, HER2-enriched, and triple negative (Kennecke et al. 2010). Screened BC has been shown to have independently lower mortality rates compared with non-screened BC (Mook et al. 2011). Finally, we also adjusted BC recurrence risk based on BC subgroup, exposure to adjuvant/palliative chemotherapy, adjuvant/palliative trastuzumab, and adjuvant/palliative endocrine therapy based on previous findings (Perez et al. 2011; Hortobagyi 1998; Mauri et al. 2006; Mouridsen et al. 2003; Mouridsen et al. 2001; Slamon et al. 2001).

Patterns of stage-specific treatments were adapted from DATASUS and from the literature. For DCIS, two treatments were possible: surgery with or without radiotherapy (Wapnir et al. 2011; Baxter et al. 2004; Meijnen et al. 2008). For invasive BC, five treatments were possible: surgery, anti-HER2 adjuvant biologic therapy, radiotherapy, chemotherapy, and endocrine therapy. The last three treatments could be used in an adjuvant as well as in a palliative setting, while anti-HER2 therapy was only allowed in adjuvant stage 2 and 3 settings for HER2-positive patients for 1 year (Perez et al. 2011). Further, the sensitivity of the mammography in the base case was adapted from Breast Cancer Surveillance Consortium (BCSC) data (Kerlikowske et al. 2011). The BCSC database was chosen because it is the largest source of effectiveness data from population screening using both film and digital technology.

The deployment of an FFDM screening program may cause higher recall rates compared with an SFM-based program (Bluekens et al. 2010). For the first round of screening in the present study (prevalence screening), recall rates were 4.29% and 3.41% for FFDM and SFM, respectively. For the second round (incidence screening), recall rates were 1.69% and 1.01% (Bluekens et al. 2010). We assumed that all recalled women would undergo another mammography and/or ultrasound. Approximately 5% and 3% of the recalled women underwent fine needle aspiration and surgical biopsy, respectively (Moss et al. 2006).

All death rates were adapted from the Brazilian Institute of Geography and Statistics Census (2010) and BC deaths were calibrated based on the Mortality Information System of Brazil (DATASUS 2000).

**Figure 2:**
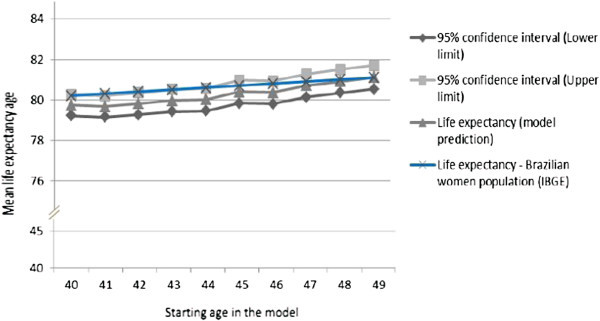
**Model predicted life expectancy and 95% confidence interval for women 40 years and older, and estimates from the Brazilian Institute of Geography and Statistics Cencus.**

We tested whether the model was calibrated according to the life expectancy of Brazilian women (IBGE 2010). The model does not include input parameters for life expectancy, which is estimated indirectly as a function of the parameters for relapse rates, progression, and overall and BC deaths. Thus, the life expectancy of Brazilian women was defined as an appropriate parameter to validate the model in Brazil. Figure 2 presents the life expectancy predicted in the model at a 95% confidence interval.

### Screening and participation rates

There is no formal screening activity in Brazil (despite some isolated initiatives at a regional level). Therefore, opportunistic screening is considered to be usual care in the Brazilian public health system. DATASUS shows that approximately 18% of women above 50 have undergone SFM (Ministério_Saúde_Brasil, DATASUS 2011). A Brazilian prospective cohort achieved a similar opportunistic screening rate of approximately 24% (Marchi & Gurgel 2010). Finally, annual participation rates ranged from 18% to 70%.

### Costs and health outcomes

Table 1 presents the costs and utilities applied in the model. Total costs consist of the costs of primary care consultancy, mammography screening, additional work-up exams (when required), cancer diagnostic procedures (images, biopsy, pathology), cancer staging (images), cancer treatment (surgery, radiotherapy, chemotherapy, anti-HER2, and endocrine therapy), and cancer follow-up. Costs were obtained from Ministério_Saúde_Brasil, DATASUS (2011) and the BC database of resource utilization in the public healthcare system in Brazil (Souza 2012). All costs are expressed in 2010 Brazilian Real (US$ 1 = R$ 1.67). Quality-adjusted life years (QALYs) were estimated based on the patient’s SF-6D scores (Souza 2012; Cruz 2010).

### Base case analysis

Using a set of natural history input parameters, we calculated the expected costs and effectiveness of each strategy in base case and sensitivity analyses. The costs and effects of each simulated screening program were then assessed. Future costs and health effects (e.g., life years and utilities losses) were discounted at a rate of 5% according to the Brazilian Guidelines for Health Technology Assessment (Ministério_Saúde_Brasil 2009). After ranking them in order of increasing costs and eliminating all dominant strategies (greater cost and fewer benefits than any other combination of strategies), we calculated incremental cost-effectiveness ratios (ICERs).

Since there is no recommended threshold to determine whether an intervention is cost-effective in Brazil (Ministério_Saúde_Brasil 2009), we adapted the recommendations of the World Health Organization, which suggests that a cost-effective intervention would avert one additional disability-adjusted life year for less than three times average per capita GDP (World Health Organization 2001). We assumed that society’s willingness to pay (WTP) for one additional disability-adjusted life year was equivalent to its WTP for one QALY. This approach has been used in previous economic evaluations performed in Brazil and in other middle-income countries (Goldie et al. 2007; Goldie et al. 2008; Vanni et al. 2010; Vanni et al. 2012). Programs that were more costly and less effective than other programs were immediately ruled out as inefficient (i.e., according to the simple dominance principle). The remaining programs constituted the frontier of efficient screening programs.

### Sensitivity analysis

To assess uncertainty in the model, one-way, scenario, and probabilistic sensitivity analyses were conducted. In one-way sensitivity analysis, the key parameters were varied using minimum and maximum values, as shown in Table 1. A probabilistic sensitivity analysis was also performed to explore joint uncertainty across parameters. By sampling the distribution of the model parameters, we generated 10,000 estimates for the costs and effects of each strategy. These estimates were plotted on a cost-effectiveness plane and cost-effectiveness acceptability curves were used to depict the level of uncertainty for the optimal strategy at different WTP thresholds for an additional QALY (Barton et al. 2008).

## Results

### Base case analysis

In the base case analysis, with a simulated cohort starting at 40 years, we found that the mean survival period (adjusted for quality) for usual care was 14,498, at a lifetime cost of R$ 2,075. All other screening strategies were associated with higher QALYs and additional costs. Table 2 presents the ICER results for the base case analysis. The discounted QALYs for the seven strategies were similar to those found in previous BC screening studies, while the differences between these strategies were small (Tosteson et al. 2008; Rojnik et al. 2008). However, there were greater differences in terms of expected lifetime costs.

**Table 2:** **Base-case incremental cost effectiveness results**

Strategy	Discounted costs (Brazilian Real)	Discounted effect (QALY)	Order of non-dominated strategies	ICER (R$/QALY)
**Strategy A - Usual care**	2,075	14,498	1	—
**Strategy B - SFM annual**	2,318	14,546	3	13,131
**Strategy C – SFM every 2 years**	2,125	14,532	2	1,509
**Strategy D – FFDM annual**	2,564	14,548		
**Strategy E – FFDM every 2 years**	2,259	14,533		
**Strategy F – FFDM (<50) and SFM (50–69) annual**	2,393	14,549	4	30,520
**Strategy F – FFDM annual (<50) and SFM (50–69) every 2 years**	2,254	14,538		

**Figure 3:**
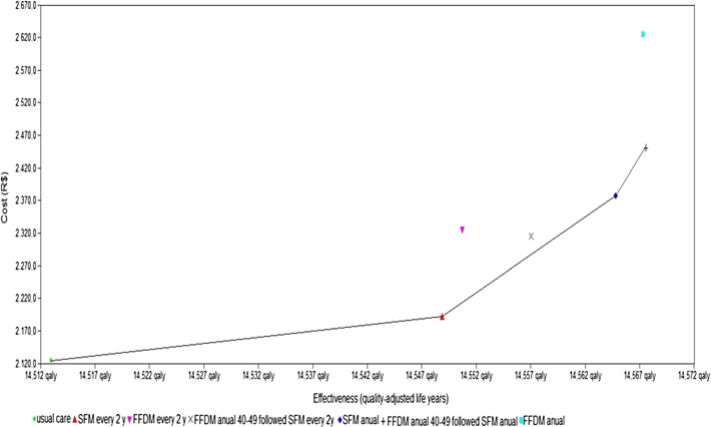
**Cost Effectiveness plane (base-case).**

Thus, the costs and effectiveness of the strategies were considered to identify which strategy represented better value for money. Figure 3 and Table 2 show that usual care (Strategy A) was the cheapest but least effective strategy. Adopting SFM every 2 years (Strategy C) was slightly more costly but also more effective, yielding an ICER of R$ 1,509 per QALY gained. The next best alternative also adopted SFM, but now annually (Strategy B), which was cost-effective at an additional R$ 13,131 per QALY gained. FFDM annual screening (Strategy E) was dominated by Strategy F, which is an age-targeted option, with FFDM used annually until 49 years and SFM annually from 50 to 69 years. For younger women (<50 years), this is the most effective strategy. With an ICER of R$ 30,520, Strategy F could also be considered to be a cost-effective strategy for an emerging country such as Brazil.

### Sensitivity analysis

In the one-way sensitivity analysis, the ranking of the seven strategies remained unchanged for most model parameters. The results were most sensitive to changes in the coverage of opportunistic screening under usual care (Strategy A). At a coverage rate of approximately 30%, SFM every 2 years showed cost savings. Although the discount rate and BC incidence seemed to play an important role in determining the magnitude of ICERs, they did not change the order of the strategies that composed the cost-effectiveness frontier.

**Figure 4:**
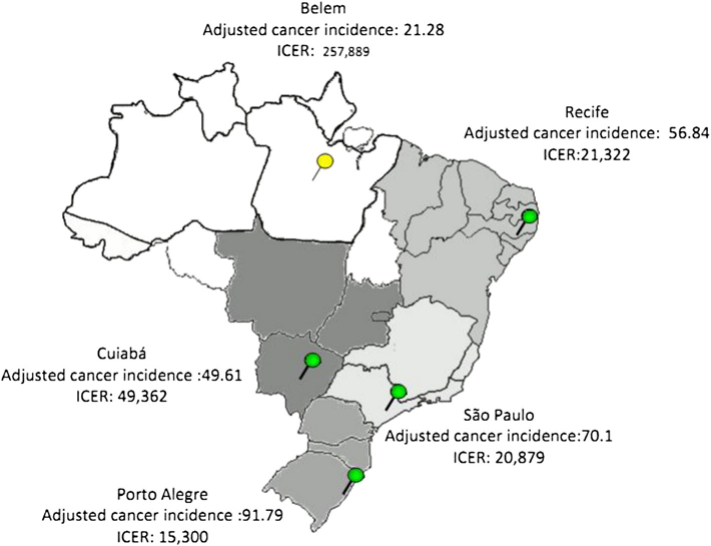
**Sensitivity analysis.** Shown is the range of the incremental cost-effectiveness ratios (ICERs) as a result of varying parameters and assumptions for screening strategy (breast cancer incidence, populational age distribution and mammography coverage).

Figure 4 presents the range of ICERs according to BC incidence, age distribution, and mammography coverage by Brazilian region (INCA 2010; MS 2011). In regions that have a lower BC incidence (e.g., Belem and Cuiaba city), ICERs have a higher probability of not being cost-effective (R$ 257,889 and R$ 49,362, respectively). On the contrary, for regions that have a higher BC incidence (e.g., São Paulo and Recife), the ICER is approximately R$ 21,000. The best scenario was in Porto Alegre, with an ICER of R$ 15,300.

**Figure 5:**
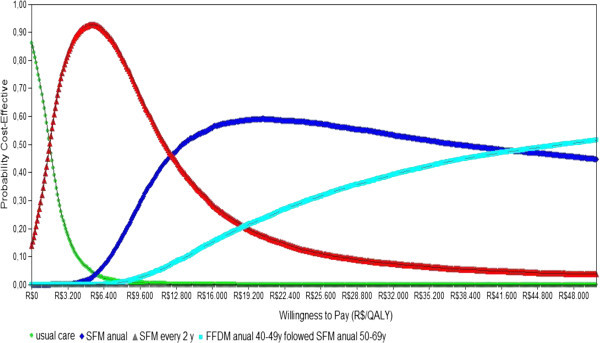
**Cost Effectiveness acceptability curve (dominated strategies not shown).**

Figure 5 reports the results of the probabilistic sensitivity analysis. By adopting the threshold suggested by the Commission for Macroeconomics in Health for cost-effectiveness interventions (R$ 17,869/QALY), and by considering both SFM strategies (annual and every 2 years), we found a high probability that SFM is a cost-effective approach for the Brazilian public health system (approximately 70% of the simulations). Moreover, at a much lower ICER of R$ 6,000/QALY, SFM every 2 years was cost-effective in more than 95% of the simulations.

Nonetheless, it is important to recognize that FFDM is increasingly used by institutions in Brazil. When we consider a cost-effective threshold of three times national GDP (R$ 53,607/QALY), we found a high probability that an age-targeted strategy (e.g., Strategy F) would be cost-effective (approximately 60% of the simulations). Considering a WTP of R$ 20,000/QALY, 10% of the simulations with Strategy F would be considered to be cost-effective. In the case of a WTP of R$ 100,000/QALY, this figure grew to 70% of the simulations.

## Discussion

BC incidence varies considerably throughout the world; indeed, age-standardized incidence is approximately fourfold higher in high-income countries in North America and Western Europe compared with countries that have a lower per capita income (Legorreta et al. 1996). A strong correlation between the age-standardized incidence of BC and average GDP per capita has been demonstrated (Lilliu et al. 2002). However, in many low- and middle-income countries, incidence is increasing faster than that in developed nations, where incidence is already high (Cody 1996).

In many Western countries, mammography screening has become the standard of care for the early detection of BC. Despite its widespread use, however, mammography is a far from a perfect means of early detection. Several limitations have been recognized, such as in the areas of false positive results, ethnic and biological differences, social and cultural barriers, and the harm-to-benefit ratio (Lu et al. 2012). Some studies have demonstrated that SFM can be cost-effective in Western countries (<US$ 50,000/QALY) (Tosteson et al. 2008; Szeto & Devlin 1996; Lindfors & Rosenquist 1995), whereas its benefit is more questionable in low- and middle-income countries (Rojnik et al. 2008; Okonkwo et al. 2008; Jakubowski et al. 1996).

Our Markov model shows that using SFM to screen for BC is a cost-effective strategy for the public health system in Brazil, a middle-income country. Considering the cost-effective threshold given by Brazil’s GDP per capita, SFM every 2 years is the strategy that has the best cost-effectiveness profile (ICER below the threshold and high probability of being cost-effective in the probabilistic sensitivity analysis). Gains in QALYs are likely to occur due to the earlier diagnostic stage of BC in women, which compensates for the additional cost of mammography screening, medical consultations, false positive results, the increase in the incidence of DCIS after the screening program has been deployed, and the over diagnosis of cancer cases. This BC treatment strategy allows for a better cure rate and lower expenses and health resource utilization (Legorreta et al. 1996; Lilliu et al. 2002; Lu et al. 2012; Butler et al. 1995; Hillner 1996; Cady 1996).

An important strength of our model is the use of the BC database, which reflects the standards of care in disease management. The base case reflects the life expectancy of Brazilian women, and QALYs were estimated from BC patients. QALYs for a healthy state were calculated based on the Brazilian population (Cruz 2010).

According to a recent government estimate, Brazil has enough mammography devices to cover more than 70% of Brazilian women aged above 50 years (MS 2011). Further, the country has deployed a program to improve the quality of mammograms in the Brazilian public health system (INCA 2009a). This adequate screening capacity along with a quality program and cost-effective data make the adoption of a national BC screening program feasible and desirable in Brazil. In this study, we demonstrate that a BC film-screening program every 2 years is a cost-effective strategy.

Regarding digital mammography screening strategies, annual screening by FFDM (Strategy E) was dominated by Strategy F (age-targeted screening). As presented in Figure 4, there was great variability in ICERs across the country, mainly related to local BC incidence. This finding suggests the importance of recognizing that for a large heterogeneous country as Brazil regionalized health policy must be considered by decision makers. For instance, in the north, digital mammography screening should not be recommended due to unfavorable cost-effectiveness. By contrast, in the southeast and south of the country, where BC incidences are higher, a digital age-targeted screening program could be considered to be a good investment in terms of value of money.

Moreover, if we consider that most mammography machines in public institutions are obsolete (>8 years old), the acquisition of replacement equipment is crucial. Thus, health policies that incentivize the acquisition of digital technology devices must be discussed for those regions with high BC incidence (mainly the southeast, south, and some areas of the northeast). In our view, this implementation should be gradual in order to minimize the budget impact from a short-term perspective, but it would allow the public system to move towards more modern technology. Although the main advantage of FFDM is seen for younger women (<50 years), other potential benefits of digitalization could be anticipated, such as the possibility of teleradiology and the more reliable retrieval of exams for future comparisons.

Our model estimates of incremental QALYs are similar to those reported in previous studies in high-income countries (Tosteson et al. 2008; Szeto & Devlin 1996; Lindfors & Rosenquist 1995), and we draw similar conclusions about the advantages of BC screening in the younger population. To the best of our knowledge, however, this is the first cost-effectiveness analysis that focuses on age-targeted digital mammography screening for women above 40 years in low- and middle-income countries and that presents a feasible strategy for an emerging country. We believe that these results can be adapted to other emerging countries with similar BC incidence rates and public healthcare structures.

In conclusion, SFM every 2 years for all women starting between the ages of 40 and 49 would be a cost-effectiveness strategy to be incorporated by the Brazilian public healthcare system. Taking into account regional specificities, age-targeted digital screening is one option to improve the outcomes of BC patients in an emerging country.
